# Angle Estimation for Range-Spread Targets Based on Scatterer Energy Focusing

**DOI:** 10.3390/s25061723

**Published:** 2025-03-11

**Authors:** Zekai Huang, Peiwu Jiang, Maozhong Fu, Zhenmiao Deng

**Affiliations:** 1 School of Electronics and Communication Engineering, Shenzhen Campus, Sun Yat-sen University, Shenzhen 518107, China; huangzk7@mail2.sysu.edu.cn (Z.H.); jiangpw@mail2.sysu.edu.cn (P.J.); 2 School of Electronics and Communication Engineering, Sun Yat-sen University, Guangzhou 510275, China; 3 Fujian Key Laboratory of Communication Network and Information Processing, Xiamen University of Technology, Xiamen 361024, China

**Keywords:** wideband radar, range-spread target, energy focusing, angle estimation

## Abstract

Wideband radar is becoming increasingly significant in modern radar systems. However, traditional monopulse angle estimation techniques are not suitable for wideband targets exhibiting range extension effects. To address this, we explore the angle estimation problem for wideband Linear Frequency-Modulated (LFM) signals and propose a new monopulse angle estimation algorithm tailored for range-spread targets. In this paper, the phase of the highest energy scatterer is used as the reference to compensate for the phases of other scatterers. The compensated scatterers are then accumulated for energy focusing. Finally, the angle of the energy-focused signal is estimated using the sum-and-difference amplitude comparison method. The proposed method can effectively focus the scatterers’ energy. Moreover, since the echo of a range-spread target can be regarded as the sum of sinusoids with different frequencies, scatterer energy focusing can effectively improve the performance of the detector. To further demonstrate the practicality of the proposed angle estimation method, it is combined with the detector to evaluate its performance. Simulation results comparing the proposed method with other approaches validate its effectiveness and demonstrate that it achieves a lower signal-to-noise ratio (SNR) threshold and higher angular accuracy. Through the proposed method, tracking and imaging can be achieved entirely within the wideband radar framework. The proposed method can also be extended to other sensor systems, advancing the development of sensor technologies.

## 1. Introduction

Radar systems are not affected by adverse environmental conditions and exhibit penetration capabilities, enabling the effective monitoring of spatial targets under any weather conditions and at any time [1]. Modern radar systems typically operate in narrowband mode to detect targets and estimate parameters such as range, velocity, and angle [2,3,4]. Velocity is measured using Doppler frequency shifts, while angle estimation is performed using traditional amplitude comparison monopulse techniques. However, narrowband radar often fails to achieve the desired accuracy in parameter estimation, and its low resolution limits target imaging and recognition. As a result, practical radar systems often alternate between narrowband and wideband waveform modes, leading to resource inefficiency [5]. Wideband radar provides richer target information than narrowband radar, offering enhanced stealth and anti-jamming capabilities. If detection and tracking could be achieved using only wideband waveforms, eliminating the need to alternate between narrowband and wideband modes, it would significantly improve radar resource utilization, reduce interception probability, and enhance system robustness against interference [6]. Consequently, wideband radar is of critical research interest for high-precision target detection and motion parameter estimation.

Given the requirements for real-time processing and implementation feasibility, modern radar systems commonly use monopulse techniques for angle measurement [7,8,9]. Monopulse techniques rely on differences in the amplitude [10,11] and the phase [12,13] of target echoes received at different antenna faces. Angle estimation requires a higher signal-to-noise ratio (SNR) than range or velocity measurements. In traditional narrowband radar, angle estimation is achieved through amplitude comparison of scatterers within the range cell containing the target. However, for range-spread targets, whose energy is distributed across multiple range cells, applying amplitude comparison only to the cell with the highest energy results in degraded estimation performance.

Furthermore, for targets with uniform energy distribution, the location of the highest energy point may shift, introducing measurement errors that adversely affect the SNR threshold and estimation accuracy. Since angle estimation errors significantly impact target tracking performance, achieving high-accuracy angle measurement is essential for effective tracking and proper operation in “all-wideband” mode. Monopulse angle estimation techniques vary among radar systems and application scenarios. Zhang et al. converted angle estimation into a frequency estimation problem, achieving unambiguous angle estimates through cross-correlation functions of echoes received at different antenna faces. However, the large ambiguity range of frequency estimation limits its accuracy [14]. Xiong et al. addressed phase ambiguity by combining phase comparison and frequency estimation techniques, refining phase differences using coarse path differences derived from frequency estimation. This approach achieves unambiguous angle estimation while mitigating the ambiguity caused by short wavelengths and large antenna separations [15]. To address the problem that interactions between multiple close targets can significantly degrade angle estimation accuracy, An et al. proposed a direction-of-arrival estimator called “Classify-Track” for radar operation in long-duration coherent integration mode [16]. In [17], amplitude-based monopulse estimation was utilized across adjacent beamports of a Rotman lens to enhance resolution. In [18], a super-resolution parameter estimation algorithm, combining wideband and narrowband processing, was introduced to improve the angular resolution of wideband monopulse radar. In addition to amplitude-based and phase-based methods, Direction-of-Arrival (DOA) estimation is also a commonly used technique for angle estimation in radar systems. DOA estimation typically relies on array antennas to receive signals from different directions, calculating the phase or time differences between various receiving points to estimate the position of the signal source. The phase difference or arrival time difference is usually closely related to the propagation path of the signal and the geometry of the array. Common DOA estimation methods include Minimum Variance Distortionless Response (MVDR), the Multiple Signal Classification (MUSIC) algorithm, and others. In recent years, there has been significant progress in the research on DOA estimation. Ref. [19] investigated the target localization and association problem under a complex multipath propagation environment. The proposed algorithm localizes spatial sources and associates incident paths with each source without requiring additional decorrelation preprocessing or prior information related to the propagation environment. Ref. [20] proposed a dictionary learning algorithm to solve the grid mismatch problem in DOA estimation using both the array sensor data and the sign of the array sensor data. Ref. [21] proposed exploiting Orbital Angular Momentum (OAM) to design circular-support continuous aperture distributions and concentric-ring arrays for target localization applications. Ref. [22] proposed a novel coupling-informed data-driven algorithm tailored for the concurrent estimation of frequency and angle within a uniform linear array (ULA) while addressing the complicating influence of mutual coupling. With the development of deep learning, there have been recent advancements in angle estimation algorithms based on deep learning [23,24].

To address the low accuracy of traditional monopulse methods for range-spread targets, a novel energy-focusing angle estimation (EFAE) method is proposed. The scatterers of range-spread targets are separated in range, but their angles are the same. Therefore, this method first takes the phase of the peak scatterer as a reference to compensate for the phases of scatterers spread across other range cells. The compensated scatterers’ signals are then accumulated to achieve energy focusing. Subsequently, the focused signal is processed using the sum-and-difference amplitude comparison method to estimate the target’s angle. The angle estimation method proposed in this paper effectively reduces the SNR threshold and improves angle estimation accuracy. Moreover, since the echo of range-spread targets can be regarded as a sum of sinusoidal waves at different frequencies [25], the energy-focusing method proposed in this paper can improve the performance of optimal sinusoidal wave detectors. To further illustrate the practicality of the proposed angle estimation method, the detector can be combined with the proposed approach, applying EFAE solely to the detected target. This integration of target detection and angle estimation has significant practical value.

The remainder of this paper is organized as follows. Section 2 presents an overview of the traditional difference-of-amplitude angle estimation methods. Section 3 introduces the signal model of range-spread targets, provides an overview of the detector, derives the energy-focusing angle estimation algorithm proposed in this paper, and analyzes the SNR. Section 4 presents the simulation results and validates the proposed method using measured data. Section 5 concludes this paper with a summary.

## 2. Conventional Monopulse Amplitude Comparison Angle Estimation

Amplitude comparison monopulse angle measurement involves summing and differencing the echoes received simultaneously by two beams pointing in different directions. After processing, the sum and difference signals are obtained, with the difference signal known as the angle-error signal. When the target is located along the equal-signal axis, the echo amplitudes received by the two beams are equal. After differencing the two signals, the feedback value is zero, indicating that the difference signal is also zero. When the target deviates from the equal-signal axis by a small angle, the error angle is proportional to the output amplitude of the difference signal, and its phase is determined by the direction of deviation. The sum signal can be used not only for range tracking and target detection but also as a phase reference for the angle-error signal.

**Figure 1:**
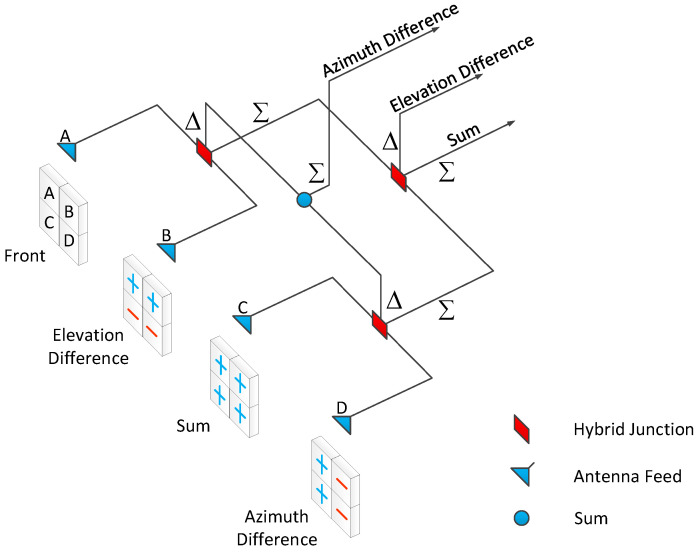
Schematic of antenna sum and difference pulses.

The echo of the target can be represented as a matrix centered on the antenna axis, as shown in Figure 1, where the four quadrants represent the four beams. When the target deviates from the antenna axis, an energy imbalance occurs between the different beams. By dividing the difference-channel voltage by the sum-channel voltage, the target’s angle can be determined [1].

## 3. Angle Estimation Based on Scatterer Energy Focusing

### 3.1. Range-Spread Target Signal Model

Linear Frequency-Modulated (LFM) signals are widely used in wideband radar systems. LFM signals increase the radio-frequency pulse width, thereby enhancing transmitted energy and maintaining a sufficiently wide frequency spectrum to ensure the radar’s range resolution. The analytical form of the LFM signal transmitted by the radar can be expressed as

(1) $$s_{t}\left(\hat{t},t_{m}\right)=\mathrm{rect}\left(\frac{\hat{t}}{T_{p}}\right)\exp\left[j2\pi \left(f_{c}t+\frac{1}{2}\gamma {\hat{t}}^{2}\right)\right]$$

(2) $$\mathrm{rect}\left(a\right)=\left\{\begin{array}{c} 1,|a|\leq 0.5\\ 0,|a|>0.5 \end{array}\right.$$

where $T_{p}$ is the pulse duration, $\hat{t}$ is the fast time, $t_{m}=mT_{r}$ is the slow time, $t=\hat{t}+t_{m}$ is the time, $T_{r}$ is the pulse repetition interval (PRl), $f_{c}$ is the signal carrier frequency, and γ is the frequency-modulation slope.

The radar echo model when the target is an ideal single-point target can be expressed as

(3) $$\begin{array}{cc} s_{i}\left(\hat{t},t_{m}\right) & =z_{i}\mathrm{rect}\left(\frac{\hat{t}-\frac{2R_{i}}{c}}{T_{p}}\right)\\ \; & \times \exp\left(j2\pi \left(f_{c}\left(t-\frac{2R_{i}}{c}\right)+\frac{1}{2}\gamma {\left(\hat{t}-\frac{2R_{i}}{c}\right)}^{2}\right)\right)+u_{i}\left(\hat{t},t_{m}\right) \end{array}$$

where $R_{i}$ is the range between the target and the radar along the radar line-of-sight direction, $z_{i}$ represents the fluctuation of the echo, and $u_{i}$ is the complex Gaussian white noise.

Pulse compression is performed on the signal using dechirp processing. Dechirp processing involves using a reference LFM signal with the same carrier frequency and frequency-modulation slope as the transmitted LFM signal but with a constant duration. This reference signal is then used for differential frequency processing against the echo. The resulting frequency-difference output is then subjected to the Fast Fourier Transform (FFT) in the fast-time domain to obtain the narrow pulse corresponding to the target’s echo in the frequency domain. Let the dechirped reference range be denoted as $R_{\mathrm{ref}}$. The reference signal is then expressed as

(4) $$\begin{array}{cc} s_{\mathrm{ref}}\left(\hat{t},t_{m}\right) & =\mathrm{rect}\left(\frac{\hat{t}-\frac{2R_{\mathrm{ref}}}{c}}{T_{\mathrm{ref}}}\right)\\ \; & \times \exp\left(j2\pi \left(f_{c}\left(t-\frac{2R_{\mathrm{ref}}}{c}\right)+\frac{1}{2}\gamma {\left(\hat{t}-\frac{2R_{\mathrm{ref}}}{c}\right)}^{2}\right)\right) \end{array}$$

where $T_{\mathrm{ref}}$ is the pulse duration of the reference signal, which should be slightly longer than the pulse duration $T_{p}$ of the transmitted signal. After mixing Equations (3) and (4), the frequency-difference output of dechirp processing is given by

(5) $$s_{\mathrm{if}}\left(\hat{t},t_{m}\right)=s_{i}\left(\hat{t},t_{m}\right)\cdot s_{\mathrm{ref}}^{*}\left(\hat{t},t_{m}\right)$$

where ${\left(\cdot \right)}^{*}$ denotes the complex conjugate operation. If the range difference of the point target relative to the reference range $R_{\Delta }=R_{i}-R_{\mathrm{ref}}$, Equation (5) for one pulse repetition cycle can be written as

(6) $$\begin{array}{cc} s_{\mathrm{if}}\left(\hat{t},t_{m}\right) & =z_{i}\mathrm{rect}\left(\frac{\hat{t}-\frac{2R_{i}}{c}}{T_{p}}\right)\\ \; & \times \exp\left(-j\frac{4\pi }{c}\gamma R_{\Delta }\hat{t}\right)\exp\left(-j\frac{4\pi }{c}f_{c}R_{\Delta }+j\frac{4\pi }{c^{2}}\gamma R_{\Delta }^{2}\right)+{\tilde{u}}_{i}\left(\hat{t},t_{m}\right) \end{array}$$

It is important to note that the signal described by Equation (6) starts at the time base corresponding to the reference range. After pulse compression, the echo of the single-point target becomes a narrowband pulse signal, where the frequency is proportional to the range difference between the point target and the range window position. The relationship is given by $f_{i}=-\gamma 2R_{\Delta }/c$. The FFT is then applied in the fast-time domain to obtain the narrow pulse in the frequency domain corresponding to the echo.

(7) $$\begin{array}{cc} S_{\mathrm{if}}\left(f_{i},t_{m}\right) & =z_{i}T_{p}\mathrm{sinc}\left(T_{p}\left(f_{i}+\gamma \frac{2R_{\Delta }}{c}\right)\right)\\ \; & \times \exp\left(-j\left(\frac{4\pi }{c}R_{\Delta }f_{i}+\frac{4\pi }{c}R_{\Delta }f_{c}+\frac{4\pi }{c^{2}}\gamma R_{\Delta }^{2}\right)\right)+{\tilde{U}}_{i}\left(f_{i},t_{m}\right) \end{array}$$

where sinc(t)=sin(πt)/πt. It can be seen from the sinc function that the frequency is proportional to the range difference between the point target and the range window. The frequency-domain resolution of the dechirped narrow pulse is $1/T_{p}$, and the range resolution is

(8) $$\rho_{r}=\frac{c(\Delta f/\gamma )}{2}=\frac{c}{2\gamma T_{p}}=\frac{c}{2B}$$

After pulse compression, the wideband radar signal, with a wide frequency sweep, is transformed into an extremely short narrow pulse. This significantly improves the range resolution, achieving sub-meter accuracy. The range distribution of the target after pulse compression is typically referred to as the High-Resolution Range Profile (HRRP).

**Figure 2:**
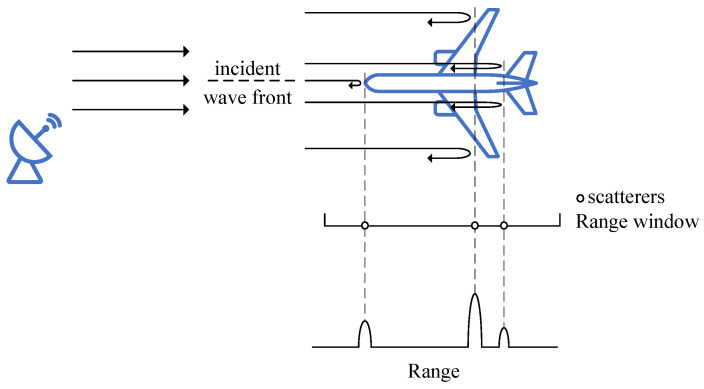
Range-spread target scatterers model.

When wideband radar is used to detect a spatial target, the radar’s range resolution is smaller than the length of the target along the radar line of sight. As a result, the echo of the target spreads in the range dimension, appearing as multiple scatterers distributed along the range axis, as shown in Figure 2.

Assuming that the target consists of *P* scatterers, the echo of a range-spread target is the vector sum of the echoes of the *P* scatterers, and the dechirped echo is expressed as

(9) $$\begin{array}{cc} s\left(\hat{t},t_{m}\right) & =\sum_{i=1}^{P}z_{i}\mathrm{rect}\left(\frac{\hat{t}-\frac{2R_{\Delta i}}{c}}{T_{p}}\right)\\ \; & \times \exp\left(-j\frac{4\pi }{c}\gamma R_{\Delta i}\hat{t}\right)\exp\left(-j\frac{4\pi }{c}f_{c}R_{\Delta i}+j\frac{4\pi }{c^{2}}\gamma R_{\Delta i}^{2}\right)+\tilde{u}\left(\hat{t},t_{m}\right) \end{array}$$

To simplify the equation, the second and third phase terms in Equation (9) are denoted as $\Phi (R_{\Delta i})$:

(10) $$\Phi \left(R_{\Delta i}\right)=\exp\left(-j\frac{4\pi }{c}f_{c}R_{\Delta i}+j\frac{4\pi }{c^{2}}\gamma R_{\Delta i}^{2}\right)$$

Equation (9) simplifies to

(11) $$s\left(\hat{t},t_{m}\right)=\sum_{i=1}^{P}z_{i}\mathrm{rect}\left(\frac{\hat{t}-\frac{2R_{\Delta i}}{c}}{T_{p}}\right)\exp\left(-j\frac{4\pi }{c}\gamma R_{\Delta i}\hat{t}\right)\Phi \left(R_{\Delta i}\right)+\tilde{u}\left(\hat{t},t_{m}\right)$$

**Figure 3:**
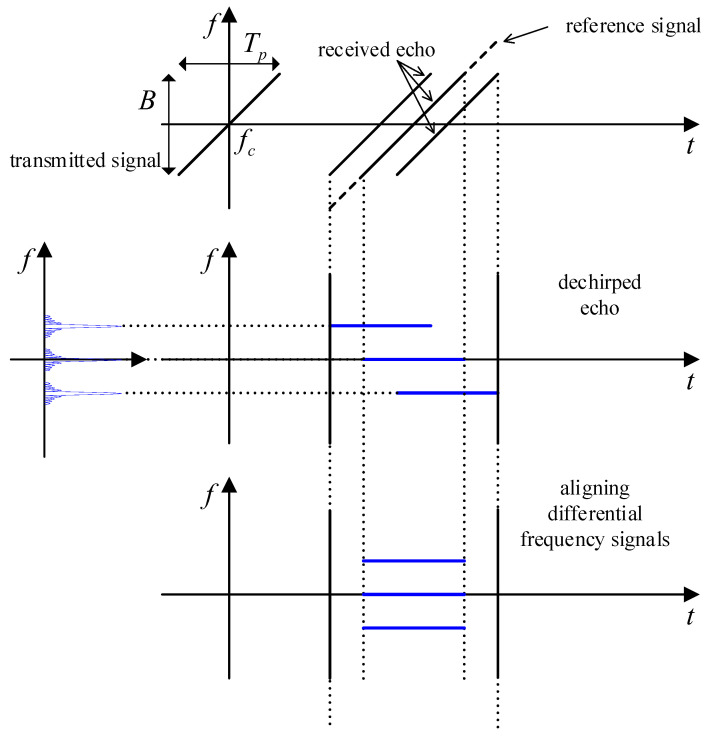
Radar transmission, reception, and dechirp process.

In practical engineering applications, the selection of a reference signal depends on the size of the target and its radial distance relative to the radar. Ideally, the reference signal should encompass the echoes from all scatterers within the range window, as shown in Figure 3. Although the echo duration remains constant, the start time of the beat-frequency signal after mixing varies for scatterers at different distances. To address this range shift in many practical scenarios, a filter is used to correct the misalignment. This filter provides a unit amplitude response across all frequencies and introduces a group delay at a specific frequency. Consequently, the signals from all scatterers are temporally aligned, facilitating subsequent data processing.

### 3.2. Theoretical Deduction

As described in Section 1, the angle estimation method proposed in this paper has the ability to focus the energy of scatterers from a range-spread target, thereby improving the performance of sinusoidal wave detectors. Since the echo of a range-spread target can be regarded as the sum of sinusoidal waves at different frequencies, energy focusing also offers advantages for detecting range-spread targets. Therefore, to further illustrate the practicality of the proposed angle estimation method, we integrate target detection and angle estimation in our study. This integration of target detection and angle estimation holds significant value in practical engineering applications.

Section 3.2.1 derives the detector used in this study, while Section 3.2.2 provides a detailed derivation of the proposed energy-focusing angle estimation method.

#### 3.2.1. Energy-Focusing Detector

Equation (11) shows that the echo of a range-spread target after pulse compression is essentially a superposition of multiple complex sinusoidal waves, where the frequency of each sinusoidal wave depends on the distance of its corresponding scatterer. Therefore, the detection of a range-spread target can be regarded as a signal detection problem for multi-component sinusoidal waves.

The scattering characteristics of a range-spread target can be inferred from the HRRP of the radar echo. Therefore, we focus on the HRRP of the target to study the detection of a range-spread target in complex Gaussian white noise. The detection of a range-spread target is modeled as a binary hypothesis testing problem, which is expressed as [26,27,28,29,30,31,32,33,34,35]

(12) $$\begin{array}{ccc} H_{0}: & \;z[n]=w[n], & n=0,1,\ldots ,N-1\\ H_{1}: & \;z[n]=s[n]+w[n], & n=0,1,\ldots ,N-1 \end{array}$$

where s[n] represents the dechirped complex signal of the range-spread echo with *P* scatterers and w[n] is the complex Gaussian white noise with a mean of 0 and variance $\sigma^{2}$. The signal s[n] can be expressed as

(13) $$s\left[n\right]=\sum_{i=1}^{P}A_{i}\exp\left(j2\pi f_{i}n+j\phi_{i}\right)=\sum_{i=1}^{P}A_{i}\cos\left(2\pi f_{i}n+\phi_{i}\right)+j\sum_{i=1}^{P}A_{i}\sin\left(2\pi f_{i}n+\phi_{i}\right)$$

where $A_{i}$, $\phi_{i}$, and $f_{i}$ denote the amplitude, phase, and frequency of the *i*-th scatterer, respectively, which are all unknown parameters. For simplicity in subsequent derivations, z[n] and s[n] are represented in terms of their real and imaginary parts:

(14) $$\begin{array}{cc} z[n] & =x[n]+jy[n]\\ s[n] & =u[n]+jv[n] \end{array}$$

where $u\left[n\right]=\sum_{i=1}^{P}A_{i}\cos\left(2\pi f_{i}n+\phi_{i}\right)$, $v\left[n\right]=\sum_{i=1}^{P}A_{i}\sin\left(2\pi f_{i}n+\phi_{i}\right)$. Since discrete complex signals can statistically be modeled as a joint distribution of their real and imaginary parts, the generalized likelihood ratio (GLR) is given by

(15) $$\begin{array}{cc} L_{G}\left(z\left[n\right]\right) & =\frac{p(z\left[n\right];{\hat{A}}_{i},{\hat{\phi }}_{i},{\hat{f}}_{i},H_{1})}{p(z\left[n\right];H_{0})}\\ \; & =\frac{\frac{1}{{\left(2\pi \sigma^{2}\right)}^{N}}\exp\left[-\frac{1}{2\sigma^{2}}\sum_{n=0}^{N-1}\left({\left(x\left[n\right]-\hat{u}\left[n\right]\right)}^{2}+{\left(y\left[n\right]-\hat{\delta }\left[n\right]\right)}^{2}\right)\right]}{\frac{1}{{\left(2\pi \sigma^{2}\right)}^{N}}\exp\left[-\frac{1}{2\sigma^{2}}\sum_{n=0}^{N-1}\left(x^{2}\left[n\right]+y^{2}\left[n\right]\right)\right]} \end{array}$$

where ${\hat{A}}_{i}$, ${\hat{\phi }}_{i}$, and ${\hat{f}}_{i}$ are the maximum likelihood estimates (MLEs) of $A_{i}$, $\phi_{i}$, and $f_{i}$, respectively. If $L_{G}\left(z\left[n\right]\right)>\gamma$ (a threshold), $H_{1}$ is accepted. As the probability density function under $H_{0}$ is independent of $f_{i}$ and non-negative, the decision condition is equivalent to

(16) $$\frac{\max_{f_{i,i=0,1,\ldots ,P}}p\left(z\left[n\right];{\hat{A}}_{i},{\hat{\phi }}_{i},{\hat{f}}_{i},H_{1}\right)}{p(z\left[n\right];H_{0})}>\gamma$$

where γ is the threshold of the detector. Equation (16) simplifies to

(17) $$\max_{f_{i,i=0,1,\ldots ,P}}\ln\frac{p(z\left[n\right];{\hat{A}}_{i},{\hat{\phi }}_{i},{\hat{f}}_{i},H_{1})}{p(z\left[n\right];H_{0})}=\ln L_{G}\left(z\left[n\right]\right)>\ln\gamma$$

By taking the logarithm of Equation (15), we obtain

(18) $$\ln L_{G}\left(x\right)=-\frac{1}{2\sigma^{2}}\sum_{n=0}^{N-1}\left({\left(x\left[n\right]-\hat{u}\left[n\right]\right)}^{2}+{\left(y\left[n\right]-\hat{v}\left[n\right]\right)}^{2}-\left(x^{2}\left[n\right]+y^{2}\left[n\right]\right)\right)$$

Let $\alpha_{i,1}=A_{i}\cos\phi_{i}$ and $\alpha_{i,2}=-A_{i}\sin\phi_{i}$. The MLEs of $\alpha_{i,1}$ and $\alpha_{i,2}$ can be obtained from Equation (19):

(19) $$\begin{array}{cc} \frac{\partial \ln p(z\left[n\right];{\hat{A}}_{i},{\hat{\phi }}_{i},{\hat{f}}_{i},H_{1})}{\partial \alpha_{i,1}} & =0\\ \frac{\partial \ln p(z\left[n\right];{\hat{A}}_{i},{\hat{\phi }}_{i},{\hat{f}}_{i},H_{1})}{\partial \alpha_{i,2}} & =0 \end{array}$$

Solving Equation (19), we obtain the MLEs of $\alpha_{i,1}$ and $\alpha_{i,2}$:

(20) $$\begin{array}{cc} {\hat{\alpha }}_{i,1} & =\frac{1}{N}\sum_{n=0}^{N-1}\left(x\left[n\right]\cos2\pi {\hat{f}}_{i}n+y\left(n\right)\sin2\pi {\hat{f}}_{i}n\right)\\ {\hat{\alpha }}_{i,2} & =\frac{1}{N}\sum_{n=0}^{N-1}\left(x\left[n\right]\sin2\pi {\hat{f}}_{i}n-y\left(n\right)\cos2\pi {\hat{f}}_{i}n\right) \end{array}$$

Then, the MLEs of u[n] and v[n] are

(21) $$\begin{array}{cc} \hat{u}\left[n\right] & =\sum_{i=1}^{P}{\hat{\alpha }}_{i,1}\cos2\pi {\hat{f}}_{i}n+\sum_{i=1}^{P}{\hat{\alpha }}_{i,2}\sin2\pi {\hat{f}}_{i}n\\ \hat{v}\left[n\right] & =\sum_{i=1}^{P}{\hat{\alpha }}_{i,1}\sin2\pi {\hat{f}}_{i}n-\sum_{i=1}^{P}{\hat{\alpha }}_{i,2}\cos2\pi {\hat{f}}_{i}n \end{array}$$

By combining Equations (19)–(21), we obtain

(22) $$\begin{array}{cc} \ln L_{G}\left(x\right) & =-\frac{1}{2\sigma^{2}}\left(-2N\sum_{i=1}^{P}\left({\hat{\alpha }}_{i,1}^{2}+{\hat{\alpha }}_{i,2}^{2}\right)+N\sum_{i=1}^{P}{\hat{A}}_{i}^{2}\right)\\ \; & =-\frac{1}{2\sigma^{2}}\left(-N\sum_{i=1}^{P}\left({\hat{\alpha }}_{i,1}^{2}+{\hat{\alpha }}_{i,2}^{2}\right)\right)\\ \; & =\frac{N}{2\sigma^{2}}\sum_{i=1}^{P}\left({\hat{\alpha }}_{i,1}^{2}+{\hat{\alpha }}_{i,2}^{2}\right) \end{array}$$

Since

(23) $$\begin{array}{cc} \sum_{i=1}^{P}\left({\hat{\alpha }}_{i,1}^{2}+{\hat{\alpha }}_{i,2}^{2}\right) & ={\left(\frac{1}{N}\right)}^{2}\sum_{i=1}^{P}\left[{\left(\sum_{n=0}^{N-1}x\left[n\right]\cos\left(2\pi {\hat{f}}_{i}n\right)\right)}^{2}+{\left(\sum_{n=0}^{N-1}x\left[n\right]\sin\left(2\pi {\hat{f}}_{i}n\right)\right)}^{2}\right]\\ \; & =\frac{1}{N}\frac{1}{N}\sum_{i=1}^{P}{\left|\sum_{n=0}^{N-1}x\left[n\right]\exp\left(-j2\pi {\hat{f}}_{i}n\right)\right|}^{2}\\ \; & =\frac{1}{N}\sum_{i=1}^{P}I\left({\hat{f}}_{i}\right) \end{array}$$

where $I({\hat{f}}_{i})$ denotes the periodogram computed at $f={\hat{f}}_{i}$.

The detector can be expressed as

(24) $$\begin{array}{c} H_{0}:\max_{f_{i,i=0,1,\ldots ,P}}\sum_{i=0}^{P}I({\hat{f}}_{i})<2\sigma^{2}\ln\gamma =\gamma^{\prime }\\ H_{1}:\max_{f_{i,i=0,1,\ldots ,P}}\sum_{i=0}^{P}I({\hat{f}}_{i})>2\sigma^{2}\ln\gamma =\gamma^{\prime } \end{array}$$

where $\gamma^{\prime }$ denotes the new threshold of the detector.

#### 3.2.2. Energy-Focusing Angle Estimation

In Equation (11), it can be observed that the extension of a wideband target appears only in the range dimension, while the angles of the scatterers are nearly identical. Although some scatterers may experience refraction due to environmental effects, their deviation is negligible under far-field conditions. Therefore, the energy of these scatterers can be focused for angle estimation. In this paper, the EFAE algorithm uses the phase of the peak scatterer as a reference to compensate for the phases of other scatterers. The compensated scatterers are then accumulated for amplitude comparison-based angle estimation, improving the angle estimation accuracy while lowering the SNR threshold. Additionally, since focusing the scatterers’ energy aids in target detection, future performance studies of this method can combine target detection, performing angle estimation only on the echo of the detected target.

**Figure 4:**
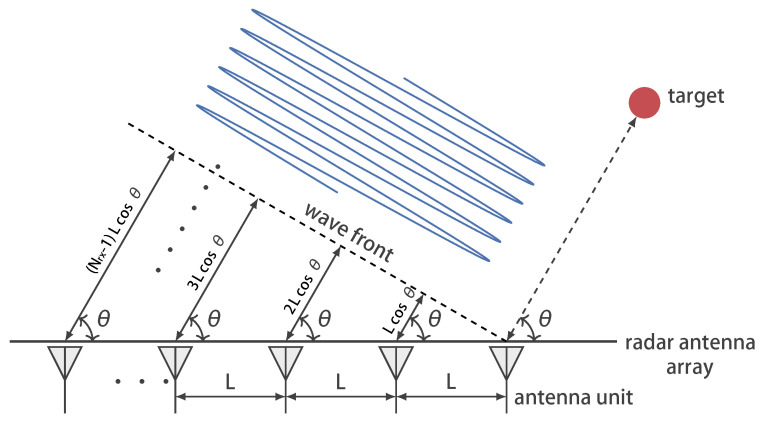
Schematic of the path difference for $N_{\mathrm{rx}}$ antenna elements under far-field conditions.

When the target’s echo arrives at the array element, a fixed range difference occurs between neighboring array elements due to their spacing. In the far-field model, all antenna elements receive the echo at the same angle of incidence, as shown in Figure 4.

$N_{\mathrm{rx}}$ denotes the number of array elements and *L* denotes the spacing between neighboring array elements. Let the beam center direction be $\phi =90^{\circ }$. According to the spatial filtering principle [36], the coefficient matrix for constructing the sum beam is expressed as

(25) $$w_{\Sigma }\left(\phi ,k\right)=\exp\left(-j\frac{2\pi (k-1)L\cos\phi }{\lambda }\right)$$

where $k\in \{1,2,\ldots ,N_{\mathrm{rx}}\}$ represents the element index. Similarly, the coefficient matrix for the difference beam is expressed as

(26) $$w_{\Delta }\left(\phi ,k\right)=\left\{\begin{array}{cc} j\exp\left(-j\frac{2\pi (k-1)L\cos\phi }{\lambda }\right) & ,k\in \{1,2,\ldots ,\frac{N_{\mathrm{rx}}}{2}\}\\ -j\exp\left(-j\frac{2\pi (k-1)L\cos\phi }{\lambda }\right) & ,k\in \{\frac{N_{\mathrm{rx}}}{2}+1,\frac{N_{\mathrm{rx}}}{2}+2,\ldots ,N_{\mathrm{rx}}\} \end{array}\right.$$

Thus, the signals of the sum and difference beams are expressed as

(27) $$\begin{array}{cc} s_{\Sigma }\left(\phi \right) & =\sum_{k=1}^{N_{\mathrm{rx}}}w_{\Sigma }\left(\phi ,k\right)\times s\left(k\right)\\ s_{\Delta }\left(\phi \right) & =\sum_{k=1}^{N_{\mathrm{rx}}}w_{\Delta }\left(\phi ,k\right)\times s\left(k\right) \end{array}$$

where s(k) represents the target echo received by the *k*-th element.

Assume that the azimuth angle of $0^{\circ }$ is parallel to the radar array plane and that the target has an azimuth angle of θ. Under these conditions, the path difference between adjacent antenna elements in the radar array is expressed as

(28) Δ=Lcosθ

Since some of the path difference is compensated for during the construction of the sum and difference beams, the remaining path difference between the antenna elements is expressed as

(29) δ=Δ−Lcosϕ

In this case, the gain of each scatterer in the sum beam is given by

(30) $$\begin{array}{cc} G_{\Sigma } & =\sum_{k=1}^{N_{\mathrm{rx}}}\exp\left(j2\pi \left(k-1\right)\frac{\delta }{\lambda }\right)\\ \; & =\frac{\sin\left(\frac{\pi N_{\mathrm{rx}}\delta }{\lambda }\right)}{\sin\left(\frac{\pi \delta }{\lambda }\right)}\exp\left(j\pi \left(k-1\right)\frac{\delta }{\lambda }\right)\\ \; & =\frac{2\sin\left(\frac{\pi N_{\mathrm{rx}}\delta }{2\lambda }\right)\cos\left(\frac{\pi N_{\mathrm{rx}}\delta }{2\lambda }\right)}{\sin\left(\frac{\pi \delta }{\lambda }\right)}\exp\left(j\pi \left(k-1\right)\frac{\delta }{\lambda }\right) \end{array}$$

The gain of the difference beam is given by

(31) $$\begin{array}{cc} G_{\Delta } & =\sum_{k=1}^{N_{\mathrm{rx}}/2}j\exp\left(j2\pi \left(k-1\right)\frac{\delta }{\lambda }\right)+\sum_{k=N_{\mathrm{rx}}/2+1}^{N_{\mathrm{rx}}}\left(-j\right)\exp\left(j2\pi \left(k-1\right)\frac{\delta }{\lambda }\right)\\ \; & =\frac{2\sin^{2}\left(\frac{\pi N_{\mathrm{rx}}\delta }{2\lambda }\right)}{\sin\left(\frac{\pi \delta }{\lambda }\right)}\exp\left(j\pi \left(k-1\right)\frac{\delta }{\lambda }\right) \end{array}$$

Hence, the peak signals of the sum and difference beams for scatterer *p* are given by $x_{p}^{\Sigma }=G_{\Sigma }A_{p}$ and $x_{p}^{\Delta }=G_{\Delta }A_{p}$, where $A_{p}$ represents the complex coefficient of the antenna array.

In Equation (9), it can be observed that the echo phases of scatterers in different range cells have different values. Phase compensation is applied to the peak signals of scatterers in other range cells for both the sum and difference beams. The range cell $R_{\Delta \max}$ of the peak scatterer is used as a reference:

(32) $$x_{p}^{\Sigma^{\prime }}=x_{p}^{\Sigma }\exp\left(-j\frac{4\pi }{c}f_{c}\left(R_{\Delta \max}-R_{\Delta p}\right)+j\frac{4\pi }{c^{2}}\gamma \left(R_{\Delta \max}^{2}-R_{\Delta p}^{2}\right)\right)$$

(33) $$x_{p}^{\Delta^{\prime }}=x_{p}^{\Delta }\exp\left(-j\frac{4\pi }{c}f_{c}\left(R_{\Delta \max}-R_{\Delta p}\right)+j\frac{4\pi }{c^{2}}\gamma \left(R_{\Delta \max}^{2}-R_{\Delta p}^{2}\right)\right)$$

where $exp\left(-j\frac{4\pi }{c}f_{c}\left(R_{\Delta \max}-R_{\Delta p}\right)+j\frac{4\pi }{c^{2}}\gamma \left(R_{\Delta \max}^{2}-R_{\Delta p}^{2}\right)\right)$ is the phase difference between each scatterer and the peak scatterer. By summing the phase-compensated signals from the scatterers, the peak signals of the sum and difference beams corresponding to the target are given by $x_{\Sigma }=\sum_{p=1}^{P}x_{p}^{\Sigma^{\prime }}$ and $x_{\Delta }=\sum_{p=1}^{P}x_{p}^{\Delta^{\prime }}$, respectively. Taking into account the noise present in practical scenarios, the peak signals of the sum and difference beams are expressed as

(34) $$y_{\Sigma }=x_{\Sigma }+\varepsilon_{\Sigma }$$

and

(35) $$y_{\Delta }=x_{\Delta }+\varepsilon_{\Delta }$$

where $\varepsilon_{\Delta }$ and $\varepsilon_{\Sigma }$ are complex noise terms that follow a Gaussian distribution.

Dividing Equation (31) by Equation (30) yields the ratio of the gains of the difference beam to the sum beam, which is expressed as

(36) $$\rho =\frac{G_{\Delta }}{G_{\Sigma }}=\tan\left(\frac{\pi N_{\mathrm{rx}}\delta }{2\lambda }\right)$$

Assuming that the SNR of the sum beam is sufficiently large, we can conclude that $G_{\Sigma }A\gg \varepsilon_{\Sigma }$. Therefore, the ratio of the peak signals of the difference beam to the sum beam is expressed as

(37) $$\begin{array}{cc} \frac{Y_{\Delta }}{Y_{\Sigma }} & =\frac{\sum_{p=1}^{P}x_{p}^{\Delta^{\prime }}+\varepsilon_{\Delta }}{\sum_{p=1}^{P}x_{p}^{\Sigma^{\prime }}+\varepsilon_{\Sigma }}\\ \; & \approx \frac{G_{\Delta }}{G_{\Sigma }}+\frac{\varepsilon_{\Delta }}{\sum_{p=1}^{P}x_{p}^{\Sigma }} \end{array}$$

where $G_{\Delta }/G_{\Sigma }$ is a real number and $\varepsilon_{\Delta }/\sum_{p=1}^{P}x_{p}^{\Sigma }$ represents complex noise. In Equation (36), it can be seen that the ratio of the gain of the difference beam to the sum beam is a real number. Therefore, in practical processing, the ratio estimation requires only the real part of the ratio between the difference- and sum-channel signals, which is expressed as

(38) ρ^=ReyΔyΣ

where Re() denotes the function that returns the real part. From Equations (36) and (38), the estimated value of the residual path difference is expressed as

(39) δ^=tan−1ReyΔyΣ2λπNrx

Substituting Equation (39) into Equation (29), the estimated path difference for the target is expressed as

(40) $$\begin{array}{cc} \hat{\Delta } & =\hat{\delta }+L\cos\phi \\ \; & =\tan^{-1}\left(\hat{\rho }\right)\frac{2\lambda }{\pi N_{\mathrm{rx}}}+L\cos\phi \end{array}$$

Substituting Equation (40) into Equation (29), the estimated azimuth angle of the target is expressed as

(41) θ^=cos−1Δ^L=cos−1tan−1ReνΔνΣ2λπNrx+LcosϕL

**Figure 5:**
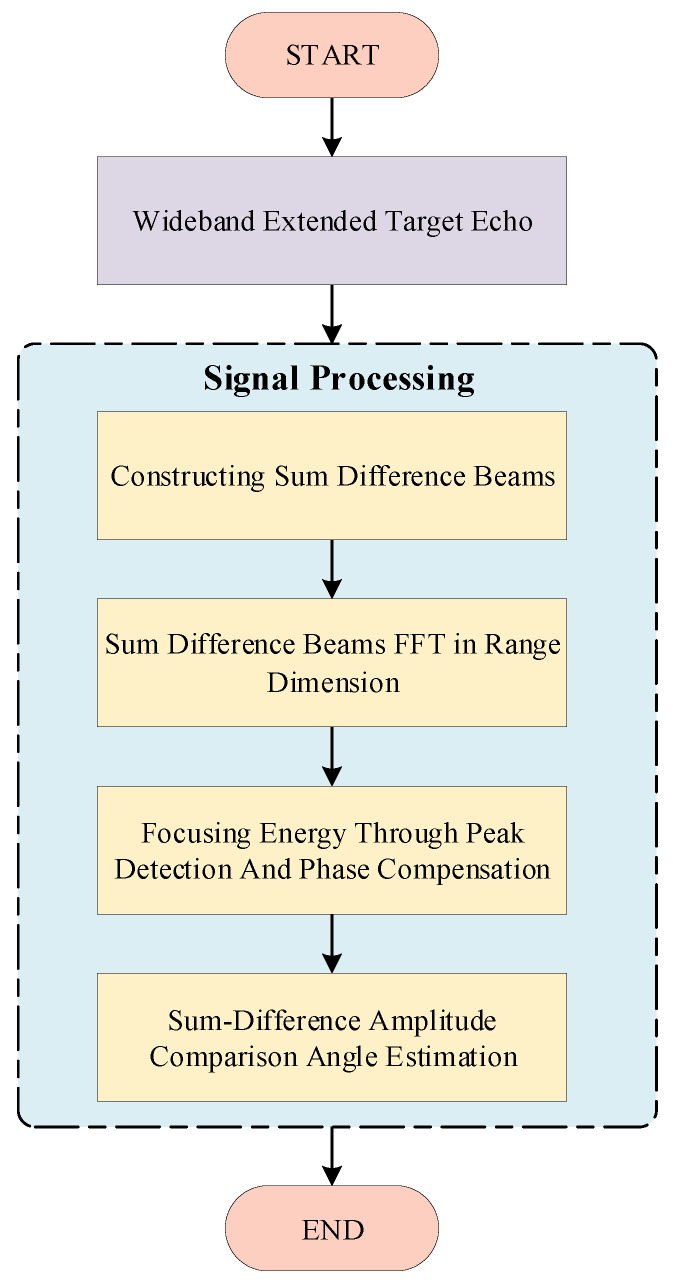
Signal-processing procedure.

The complete signal-processing flowchart is presented in Figure 5.

Here, we present the complexity analysis.

The traditional sum-and-difference amplitude method mainly involves constructing the sum and difference beam signals, applying the FFT to find the peak points, and then calculating the amplitude ratio. Assuming that the number of array elements is *M* and the number of signal sampling points is $N_{s}$, constructing the sum and difference beams requires weighting and summing across all array element channels, which has a time complexity of $O(MN_{s})$. In most practical scenarios, the number of radar array elements is relatively small and can be treated as a constant, so the time complexity is $O(N_{s})$. The time complexity of the FFT is $O(N_{s}logN_{s})$, the time complexity of finding the peak points in the sum and difference beams is $O(N_{s})$, and the time complexity of calculating the amplitude ratio to determine the angle is O(1). Therefore, the overall time complexity of the traditional sum-and-difference amplitude method is $O(N_{s}logN_{s})$.

The EFAE method proposed in this paper builds upon the sum-and-difference amplitude method by adding the steps of locating extended scatterers, performing phase compensation, and accumulating coherent signals. Given that the number of scatterers is known, the time complexity of locating the scatterers is $O(N_{s})$, and both phase compensation and signal accumulation have a constant time complexity of O(1). Therefore, the time complexity of the EFAE method is also $O(N_{s}logN_{s})$. In other words, the EFAE method only adds a certain amount of computational load but does not increase the overall complexity.

### 3.3. Detector Performance Analysis

Assume that the frequency estimation is accurate. The periodogram in Equation (23) can be rewritten as $\sum_{i=1}^{P}I(f_{i})=\sum_{i=1}^{P}\left(\xi_{i,1}^{2}+\xi_{i,2}^{2}\right)$, where

(42) $$\begin{array}{cc} \xi_{i,1} & =\frac{1}{\sqrt{N}}\sum_{n=0}^{N-1}\left(x\left[n\right]\cos2\pi f_{i}n+y\left[n\right]\sin2\pi f_{i}n\right)\\ \xi_{i,2} & =\frac{1}{\sqrt{N}}\sum_{n=0}^{N-1}\left(x\left[n\right]\sin2\pi f_{i}n-y\left[n\right]\cos2\pi f_{i}n\right) \end{array}$$

where $\xi_{i,1}$ and $\xi_{i,2}$ represent linear transformations of x[n] and y[n], which follow a joint Gaussian distribution. Under hypothesis testing, the random variables are independent under $H_{0}$ and $H_{1}$. Under $H_{0}$, $L_{G}\left(x\right)$ follows a central chi-square distribution, while under $H_{1}$, $L_{G}\left(x\right)$ follows a non-central chi-square distribution. When normalizing the periodogram $I\left(f\right)/\left(\sigma^{2}/2\right)$ and the target consists of *P* spread scatterers, the degrees of freedom are ν=2P. Consequently, $L_{G}\left(x\right)$ follows an $\chi_{\nu }^{2}$ distribution under $H_{0}$ and an $\chi_{\nu }^{\prime 2}\left(\lambda \right)$ distribution under $H_{1}$, where the non-central parameter is given by

(43) $$\begin{array}{cc} \lambda & =\sum_{i=1}^{P}\left[{\left(\frac{\sqrt{N}A_{i}\cos\phi }{\sigma /\sqrt{2}}\right)}^{2}+{\left(\frac{-\sqrt{N}A_{i}\sin\phi }{\sigma /\sqrt{2}}\right)}^{2}\right]\\ \; & =N\sum_{i=1}^{P}\frac{2A_{i}^{2}}{\sigma^{2}} \end{array}$$

The radar false alarm probability is expressed as

(44) PFA=Pr∑i=1PI(fi)>γ′;H0=Pr∑i=1PI(fi)σ2σ222>γ′σ2σ222;H0=Qχν22γ′σ2

where $Q_{\chi^{2}}\left(x\right)$ is the right-tail probability function

(45) $$Q_{\chi^{2}}\left(x\right)=\int_{x}^{\infty }p(t)dt,x>0$$

From Equation (44), the detection threshold can be obtained as

(46) $$\gamma^{\prime }=\frac{\sigma^{2}}{2}Q_{\chi_{\nu }^{2}}^{-1}\left(P_{FA}\right)$$

The detection probability is then given by

(47) PD=Pr∑i=1PI(fi)>γ′;H1=Pr∑i=1PI(fi)σ2σ222>γ′σ2σ222;H1=Qχν′2(λ)2γ′σ2

which can be further expressed as

(48) $$P_{D}=Q_{\chi_{\nu }^{\prime 2}\left(\lambda \right)}\left(Q_{\chi_{\nu }^{\prime 2}\left(\lambda \right)}^{-1}\left(P_{FA}/N_{s}\right)-\sqrt{\lambda }\right)$$

where $N_{s}$ is the signal detection window length.

### 3.4. Signal-to-Noise Ratio Analysis

**Figure 6:**
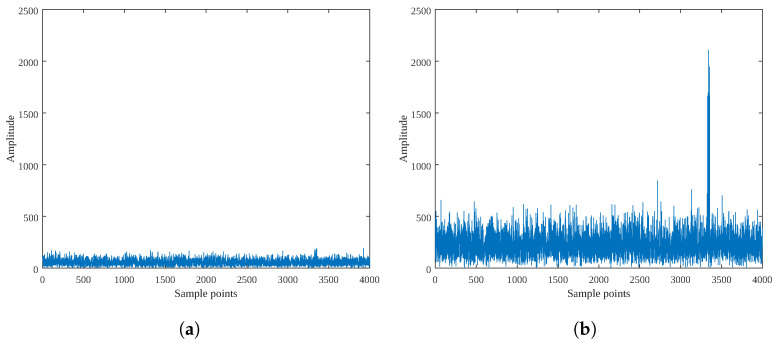
Signal-to-noise ratio analysis. (**a**) The spectrum of the echo at SNR −20 dB; (**b**) The spectrum of the sum beam at SNR −7.93 dB.

Unlike narrowband echoes, the noise of wideband echo has a broader bandwidth. The SNR for wideband echo is defined as in [37,38,39,40,41,42]. The SNR of the wideband echo is defined as $SNR_{0}=-20$ dB, and its signal spectrum is shown in Figure 6a.

The theoretical SNR gain achieved by constructing the sum beam is $10*\log10(N_{\mathrm{rx}})$ dB. Assuming that $N_{\mathrm{rx}}=16$, the SNR of the sum beam is $SNR_{1}=10*\log10\left(N_{\mathrm{rx}}\right)+SNR_{0}$ dB. The sum beam spectrum is shown in Figure 6b.

At this point, the noise power is calculated as $P_{\mathrm{noise}}=15.877$, and the signal power is $P_{\mathrm{sig}}=2.560$. Thus, the SNR at this point is $10*\log10\left(P_{\mathrm{noise}}/P_{\mathrm{sig}}\right)=-7.93$ dB, and the gain is $\left(-7.93\right)-SNR_{0}=12.07$ dB, which is consistent with the theoretical gain 10∗log10(16)=12 dB.

The amplitude used in the sum-and-difference amplitude comparison method is estimated from the spectral peak of the sum and difference beams. The SNR gain achieved by the FFT is $10*\log10(N_{s})$, where $N_{s}$ is the number of fast-time sampling points. The EFAE method fully concentrates the energy of scatterers distributed across different range cells and then performs angle estimation using the sum-and-difference amplitude comparison method. Therefore, its SNR gain is $10*\log10\left(N_{\mathrm{rx}}*N_{s}\right)+SNR_{0}$ dB.

### 3.5. Root Mean Square Error

The Cramer–Rao Lower Bound (CRLB) reveals the minimum value of the estimation variance for an unbiased estimator, serving as an important reference for evaluating the performance of an algorithm. This section presents the derivation of the CRLB for the EFAE algorithm.

Assume that the beam center direction is at 90°, and according to Equation (36), the relationship between the target’s off-boresight angle and the ratio of the sum-channel and difference-channel gains is given by

(49) $$f\left(\theta \right)=\frac{G_{\Delta }\left(\theta \right)}{G_{\Sigma }\left(\theta \right)}=\tan\left(\frac{\pi N_{\mathrm{rx}}L}{2\lambda }\sin\theta \right)$$

When the target’s off-boresight angle is within the linear region of the trigonometric function, the above equation is approximated as

(50) $$f\left(\theta \right)\approx \tan\left(\frac{\pi N_{\mathrm{rx}}L}{2\lambda }\theta \right)\approx \frac{\pi N_{\mathrm{rx}}L}{2\lambda }\theta =\eta \theta$$

where $\eta =\pi N_{\mathrm{rx}}L/2\lambda$ is the monopulse amplitude slope. Assume that the sum-channel signal is represented as

(51) $$\Sigma =s_{\Sigma }+n=\sum_{p=1}^{P}A_{p}+e_{r}+ie_{i}$$

The difference-channel signal is represented as

(52) $$\Delta =s_{\Delta }+n=\eta \theta \sum_{p=1}^{P}A_{p}+e_{r}+ie_{i^{+}}$$

where $n=e_{r}+ie_{i}$ is complex noise, and $e_{r}$ and $e_{i}$ follow a Gaussian distribution $N\left(0,\sigma^{2}/2\right)$. Assuming that the SNR is sufficiently high, then

(53) $$\frac{\Delta }{\Sigma }=\frac{\Delta }{\sum_{p=1}^{P}A_{p}+e_{r}+ie_{i}}\approx \frac{\Delta }{\sum_{p=1}^{P}A_{p}}=\eta \theta +\frac{e_{r}}{\sum_{p=1}^{P}A_{p}}+i\frac{e_{i}}{\sum_{p=1}^{P}A_{p}}$$

According to Equation (41), the estimated off-axis angle is

(54) $$\hat{\theta }=\frac{1}{\eta }\times \mathrm{re}\left(\frac{\Delta }{\Sigma }\right)\approx \frac{1}{\eta }\times \left(\eta \theta +\frac{e_{r}}{\sum_{p=1}^{P}A_{p}}\right)=\theta +\frac{e_{r}}{\eta \sum_{p=1}^{P}A_{p}}\omega$$

The definition of the SNR is

(55) $$\mathrm{SNR}=\frac{{\left(\sum_{p=1}^{P}A_{p}\right)}^{2}}{\sigma^{2}}$$

From the noise variance in Equation (54), the variance of the angle estimate is $1/2\eta^{2}\mathrm{SNR}$. So, the approximate CRLB is

(56) $$\mathrm{CRLB}=\frac{1}{2\eta^{2}\mathrm{SNR}}$$

## 4. Simulation Results

In this section, we simulate the echo of a range-spread target to perform angle estimation and present the root mean square error (RMSE) simulation results for angle estimation after detection using the proposed method. These results validate the effectiveness and advantages of the proposed EFAE method. Additionally, we compare the proposed EFAE algorithm with other angle estimation methods. The first method is the traditional sum-and-difference amplitude comparison (SDAC) method, which estimates the angle based on the scatterer with the highest energy. The second method is the Weighted Averaging Angle Estimation (WAAE) method, which performs angle estimation for all scatterers using the SDAC method. The results are then weighted and averaged based on the amplitude to obtain the target angle. The third method is the high-precision angle estimation (HPAE) method based on phase ambiguity resolution proposed in [15]. To ensure a fair comparison, all angle estimation methods are evaluated in conjunction with the detector. We compare the performance of the proposed EFAE method with these methods under different SNR conditions. The comparison demonstrates the proposed method’s advantages in angle estimation accuracy and SNR threshold. Next, we analyze the impact of the number of spread scatterers and the target off-axis angle on the EFAE method, further exploring its applicable scenarios. Finally, we analyze the performance of the proposed algorithm under different types of targets. At the end of this section, we validate the algorithm proposed in this paper using radar data collected from a commercial aircraft.

**Figure 7:**
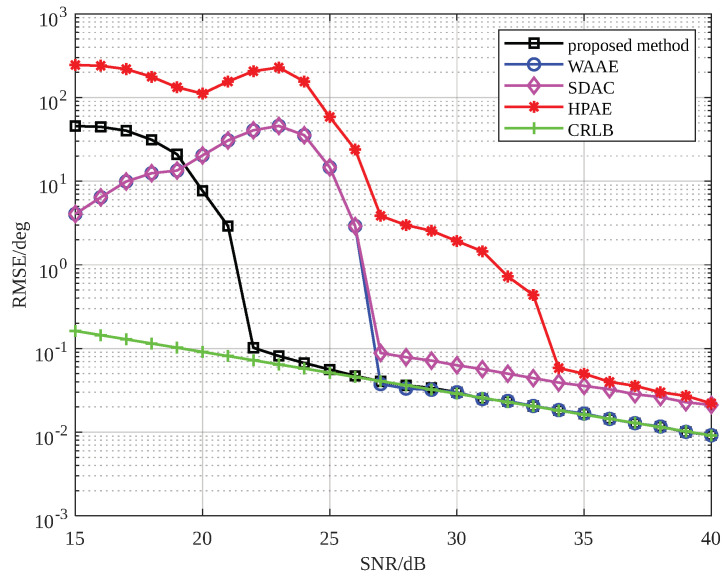
The SNR-RMSE plot of different angle estimation methods.

The simulation parameters for the radar target detection scenario are listed in Table 1. The radar carrier frequency is $F_{c}=8.75$ GHz, the signal bandwidth is B=1 GHz, the pulse duration is T=100 µs, and the sampling rate is $F_{s}=40$ MHz. The beam center direction is $\phi =90^{\circ }$. The number of scatterers of the far-field target is P=10, the target length is approximately D=4 m, the target range is R=500 m, and the target azimuth angle is $\theta =88^{\circ }$. The detector’s false alarm probability is $P_{\mathrm{fa}}=1\times 10^{-4}$. All results were obtained using 1000 Monte Carlo simulations. To facilitate comparison with the method in [15], the number of array elements is set to $N_{\mathrm{rx}}=2$, and the baseline length is set to L=10λ. The simulation results are shown in Figure 7.

In Figure 7, it is evident that at a sufficiently high SNR, both the proposed method and the WAAE method outperformed the other two methods in terms of accuracy, with the two approaches yielding comparable precision. However, compared to the WAAE method, the proposed method achieved a lower SNR threshold, indicating that the method proposed in this paper can achieve relatively high goniometric accuracy at a lower SNR. The HPAE method in [15] exhibited average performance because it is more suitable for detection scenarios with specific antenna spacing requirements, where angle ambiguity cannot be avoided. It is clear that the method proposed in this paper is better suited for common radar detection scenarios and is more practical.

**Figure 8:**
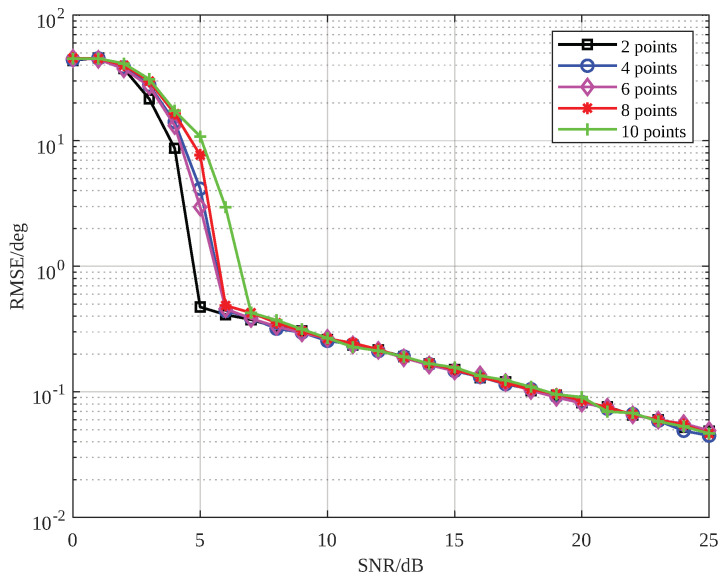
The SNR-RMSE plot of the proposed method under varying numbers of scatterers.

The highlight of the EFAE method lies in its ability to focus the energy of a target spread across different range cells, which makes the number of scatterers in a range-spread target a key factor influencing its performance. Therefore, we analyzed the impact of the number of spread scatterers on the proposed method. To obtain more distinct curve trends and a larger unambiguous angle range, the number of array elements $N_{\mathrm{rx}}$ was set to 16, and the baseline length *L* was set to λ/2. The number of scatterers was set to 2, 4, 6, 8 and 10. The simulation results are shown in Figure 8. As seen in Figure 8, as the number of scatterers increased, the SNR threshold of the proposed method gradually rose. This is because, at the same SNR, a higher scatterer count reduced the energy per point, leading to deviations in locating scatterers based on amplitude, thereby decreasing angle estimation accuracy.

**Figure 9:**
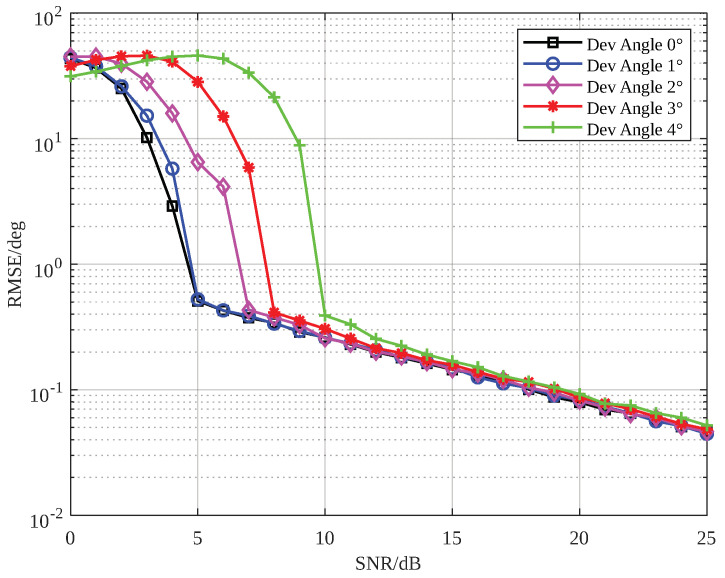
The SNR-RMSE plot of the proposed method under different deviation angles.

Next, we analyzed the effect of target deviation from the beam center direction on the proposed method. The number of scatterers was set to 10, and the deviation angles from the beam center direction were $0^{\circ }$, $1^{\circ }$, $2^{\circ }$, $3^{\circ }$, and $4^{\circ }$. The simulation results are shown in Figure 9. It is clear that as the deviation angle increased, the SNR threshold of the proposed method became higher. This is because larger deviation angles reduced the accuracy of the angle estimation based on sum-and-difference amplitude comparison.

The number of scatterers and the target’s deviation angle from the beam center direction do not affect the accuracy of the EFAE method under high SNR conditions.

**Figure 10:**
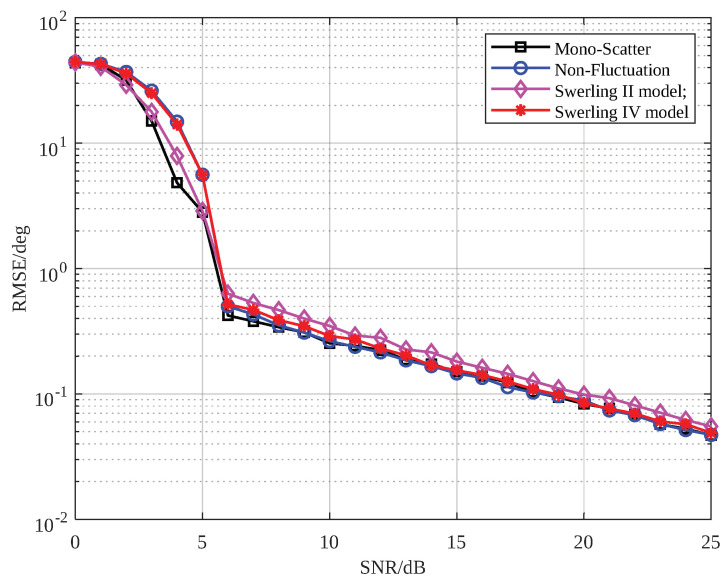
The SNR-RMSE plot of the proposed method under different types of targets.

To evaluate the robustness of the proposed method, we analyzed its performance under various target types. Four representative target types were considered: a single scatterer target, a nonfluctuating target with 10 scatterers, a Swerling II target with 10 scatterers, and a Swerling IV target with 10 scatterers. The reason for excluding Swerling I and Swerling III types is that, in the monopulse case, Swerling I and Swerling III are identical to Swerling II and Swerling IV, respectively. The simulation results, shown in Figure 10, indicate that the algorithm performed similarly across all four types of targets, demonstrating that the EFAE algorithm is suitable for common radar target models. Furthermore, the algorithm’s performance under the single-scatterer model was nearly identical to that under the multi-scatterer model, further highlighting the method’s advantages in energy focusing.

At the end of this section, the proposed angle estimation method is validated using wideband echoes from civil aviation aircraft collected by an actual radar and compared with reference methods. The radar parameters are shown in Table 2.

**Figure 11:**
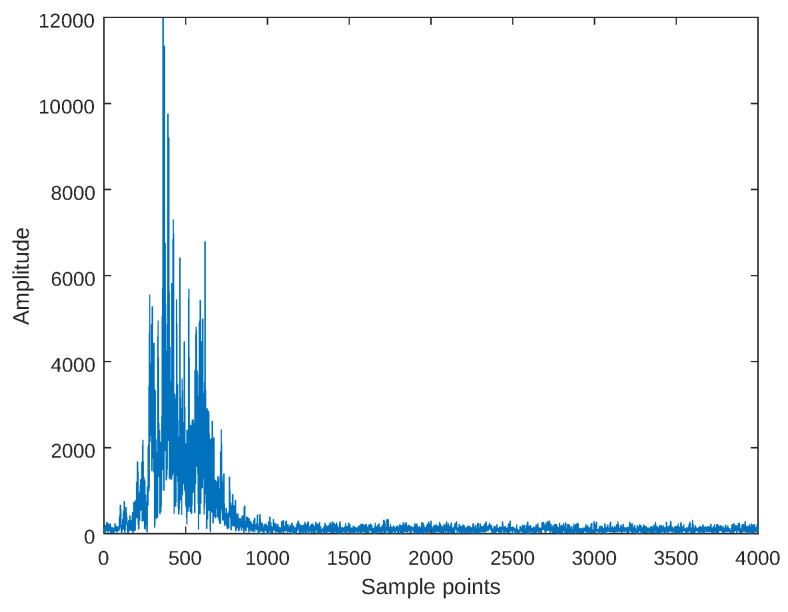
HRRP of the measured aircraft echoes.

**Figure 12:**
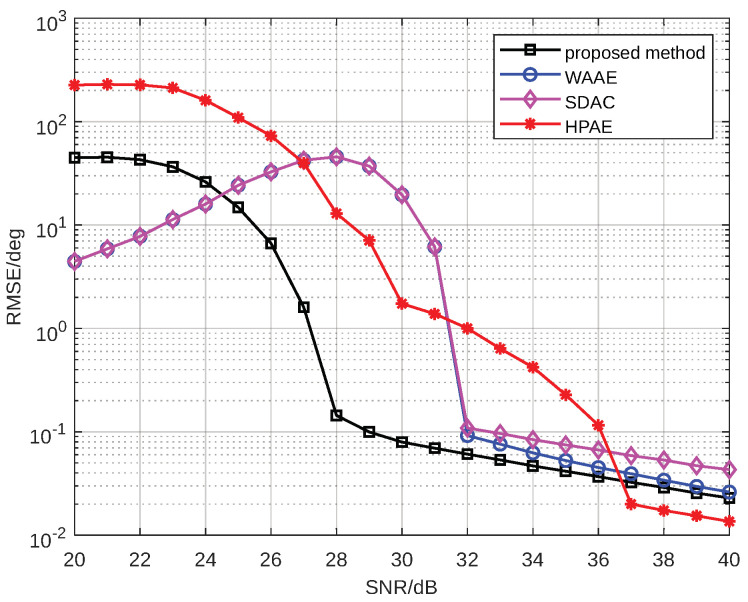
The SNR-RMSE plot of the measured aircraft echoes.

Figure 11 shows the HRRP of the aircraft echo data. The number of scatterers is unknown but assumed to be 20. During the Monte Carlo experiments, the original data with a high SNR were treated as noise-free, and complex noise was added to control its SNR for the experiment. Figure 12 presents the RMSE of the angle estimation results under different SNR conditions. The experimental results demonstrate that the EFAE method has a lower SNR threshold while maintaining relatively high angle estimation accuracy, further validating the effectiveness and advantages of the EFAE method.

Simulations validate that, compared to other methods, the proposed method achieved higher angle estimation accuracy and a lower SNR threshold, demonstrating its effectiveness. Although the SNR threshold increased with a higher scatterer count and larger deviation angles, the proposed method retained its advantages over other methods. By using this method, tracking and imaging can be performed solely with wideband radar, effectively enhancing data rates and providing improved anti-jamming capabilities.

## 5. Conclusions

In this paper, we investigate the angle estimation problem for range-spread targets in wideband radar systems. Due to the high range resolution of wideband radar, target energy is dispersed across different range cells, making traditional narrowband angle estimation methods unsuitable. To address this issue, we propose an angle estimation method based on scatterer energy focusing. This approach focuses the dispersed target energy across different range cells to reduce the SNR threshold and improve the angle estimation accuracy, enabling effective detection and high-precision angle estimation of range-spread targets in wideband radar. The algorithm is derived theoretically, validated through simulations, and compared with other methods. The results demonstrate the algorithm’s effectiveness, showing that it efficiently accumulates the energy of spread scatterers and performs better under low SNR conditions. The proposed method is significant for the angle estimation of range-spread targets in wideband radar systems.

## Figures and Tables

**Table 1:** Simulation parameters.

Symbol	Quantity	Value
$F_{c}$	Carrier frequency	8.75 GHz
*B*	Signal bandwidth	1 GHz
*T*	Pulse duration	100 µs
$F_{s}$	Sampling rate	40 MHz
ϕ	Beam center direction	$90^{\circ }$
*P*	Number of scatterers	10
*D*	Target length	4 m
*R*	Target range	500 m
θ	Azimuth angle	$88^{\circ }$
$P_{\mathrm{fa}}$	False alarm probability	$1\times 10^{-4}$
*M*	Monte Carlo simulations	1000

**Table 2:** The main system parameters of radar.

Symbol	Quantity	Value
$F_{c}$	Carrier frequency	9.5 GHz
*B*	Signal bandwidth	1 GHz
*T*	Pulse duration	200 µs
$F_{s}$	Sampling rate	20 MHz
$N_{s}$	Number of samples per chirp	4000
$N_{C}$	Number of chirps	1

## Data Availability

The original contributions presented in this study are included in the article. Further inquiries can be directed to the corresponding authors.
